# Telomeres: Implications for Cancer Development

**DOI:** 10.3390/ijms19010294

**Published:** 2018-01-19

**Authors:** Aina Bernal, Laura Tusell

**Affiliations:** Unitat de Biologia Cel·lular, Facultat de Biociències, Universitat Autònoma de Barcelona, 08193 Cerdanyola del Vallès, Spain; aina.bernal@uab.cat

**Keywords:** telomere-dysfunction, chromosome instability, cancer

## Abstract

Telomeres facilitate the protection of natural ends of chromosomes from constitutive exposure to the DNA damage response (DDR). This is most likely achieved by a lariat structure that hides the linear telomeric DNA through protein-protein and protein-DNA interactions. The telomere shortening associated with DNA replication in the absence of a compensatory mechanism culminates in unmasked telomeres. Then, the subsequent activation of the DDR will define the fate of cells according to the functionality of cell cycle checkpoints. Dysfunctional telomeres can suppress cancer development by engaging replicative senescence or apoptotic pathways, but they can also promote tumour initiation. Studies in telomere dynamics and karyotype analysis underpin telomere crisis as a key event driving genomic instability. Significant attainment of telomerase or alternative lengthening of telomeres (ALT)-pathway to maintain telomere length may be permissive and required for clonal evolution of genomically-unstable cells during progression to malignancy. We summarise current knowledge of the role of telomeres in the maintenance of chromosomal stability and carcinogenesis.

## 1. Telomere Structure

**Figure 1:**
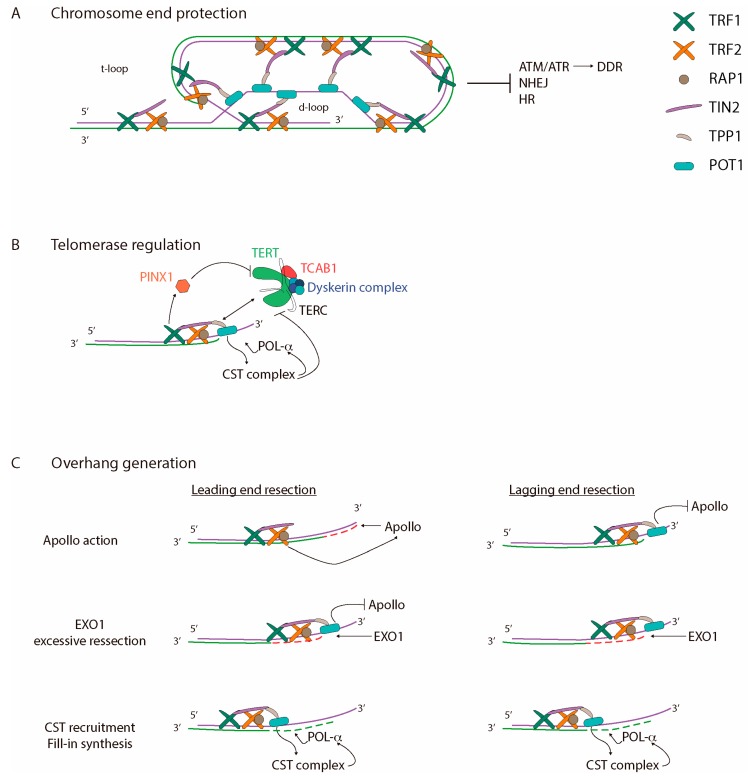
Telomere structure and maintenance. Human telomeres are specialised nucleoprotein structures that cap the end of chromosomes. Telomeric DNA is coated by shelterin proteins that regulate (**A**) chromosome end protection; (**B**) telomerase regulation; and (**C**) overhang generation. (**A**) Telomeres adopt a t-loop structure that prevents DNA damage response (DDR), by blocking ATM and ATR activation, and DNA repair activities via NHEJ and HR; (**B**) telomerase action is tightly regulated and only acts at telomeres during S-phase. Telomerase preferentially acts in short telomeres, maybe by indirect inhibition through TRF1 and PINX1 and/or other unknown mechanisms. TPP1 physically interacts with telomerase, and is essential for telomerase recruitment together with TIN2. It has been suggested that POT1-TPP1 is implicated in telomerase-telomere engagement. Besides that, POT1-TPP1 stimulates telomerase action until a certain threshold of telomeric repeats is reached. Once telomerase is blocked by CTC1-STN1-TEN1 (CST) and/or POT1, CST facilitates fill-in synthesis of the C-strand by polymerase-α (POL-α) recruitment; (**C**) Mammalian chromosomes terminate in a 3′ G-rich overhang that is essential for t-loop formation. DNA replication originates blunt ends in the leading strands, and non-blunt ends in the lagging strands. TRF2 recruits Apollo that resects 5′ strand from leading ends (dashed red line) to generate a 3′ overhang. Then, EXO1 excessively resects the leading and lagging strands (dashed red lines) to generate longer overhangs. In addition, finally, POT1 facilitates the 5′-strand fill-in (dashed green lines) by the recruitment of POL-α, through the CST complex.

The first evidence of telomeres, specialised nucleoprotein structures that protect the end of chromosomes, was observed in flies and maize [1,2]. After irradiation, broken chromosomes were easily fused to each other, driving genomic reorganizations, whereas the chromosome ends were protected from such events [1,2]. These experiments thus argued for specific characteristics of chromosome termini. In line with this, telomeres are composed by a guanosine rich conserved DNA that varies in length, sequence, and number of repeats between organisms. Mammalian telomeres are organised as repetitive TTAGGG sequences [3,4] that terminate in a single-stranded G-rich 3′ overhang [5,6]. In humans, the length of the double-stranded (ds) telomere track is around 9–15 kb, and the single-stranded (ss) DNA protrudes a few hundred nucleotides [7,8]. Telomeric DNA is coated by a six-protein complex known as the telosome, or shelterin (Figure 1A) [9]. This group of proteins specifically bind to telomeres throughout the cell cycle and function as a platform that recruits players from diverse pathways to the telomeres for maintenance and protection. Three shelterins bind directly to telomeric DNA through sequence recognition. TRF1 (Telomeric Repeat-binding Factor 1) and TRF2 (Telomeric Repeat-binding Factor 2) bind to ds-DNA [10,11,12], while POT1 (Protection Of Telomeres 1) binds to ss-DNA [13,14]. The other three proteins interact with telomeres through protein-protein interactions. TIN2 (TRF-Interacting Nuclear protein 2) links TRF1 and TRF2 proteins [15], and also interacts with TPP1 (Tripeptidyl-peptidase 1), which at the same time, binds to POT1 [16]. Therefore, TIN2 consolidates TRF1-TRF2 and POT1-TPP1 interactions at telomeric DNA [15,16]. Finally, RAP1 interacts exclusively with TRF2 [17,18,19] and DNA through structure recognition, but not through sequence preference [20].

One of the main functions of shelterins is to remodel the linear telomeric DNA to form a lariat structure that differentiates the chromosome end from a double-strand break (DSB). This arrangement is achieved through the invasion and pairing of the G-rich 3′ overhang into the preceding C-strand of the ds-telomeric tract while the telomeric G-strand is displaced. As a result, a displacement loop (d-loop) and a telomeric loop (t-loop) are formed (Figure 1A) [21]. Several studies point out the involvement of TRF2 with the generation of DNA loops in vitro [21,22,23], as lariat structures are not observed when TRF2 is absent [23]. The ability of TRF2 to generate t-loops lies in its capacity in modifying DNA topology by inducing positive supercoiled structures [24,25], as well as its ability to stimulate telomeric invasion [24,26] in a non-DNA-sequence specific manner [27]. Moreover, molecular dynamic simulation studies suggest that TRF2 proteins possess the ability of folding chromatin into a globular structure where DNA is compacted [28]. In agreement with this observation, super-resolution microscopy analysis has shown that human telomeres, in vivo, form hyper-compact globular chromatin structures through specific protein-protein and protein-DNA interactions between shelterin subunits and telomeric DNA that is essential for end protection. Indeed, depletion of shelterin components leads to telomeric chromatin decompaction that triggers access of DNA damage response (DDR) signals at telomere ends [29]. These data underscore a more complex picture for telomere structure organisation and end protection.

## 2. Maintenance of Telomere Length

Telomeres are highly dynamic structures [30] that remain in a closed-state during most cell cycle phases to protect chromosome ends. However, during S-G2 phases, telomeres become open to allow complete DNA replication and to provide a substrate for the addition of telomere repeats. Telomere elongation relies mainly on telomerase [31,32], a large ribonucleoprotein composed of an RNA template (TERC: telomerase RNA component) [33], a catalytic domain (TERT: telomerase reverse transcriptase) [34,35], the dyskerin complex composed of dyskerin-NOP10-NHP2-GAR1 [36], and TCAB1 [37]. It has been determined that telomerase assembly and maturation occurs at Cajal bodies [38,39]. However, coilin-deficient or TCAB1-deficient human cells do not present telomerase biogenesis and/or activity defects [40,41]. During cell cycle, telomerase can be located at Cajal bodies, or elsewhere in the nucleoplasm [42,43], except during S-phase when assembled telomerase is transported to telomeres [44]. Then, telomere elongation takes place shortly after duplex DNA replication is completed, and after the G-rich overhang has been processed [45]. Several mechanisms are involved in telomerase recruitment, engagement, action and disassembly.

Telomerase recruitment to the chromosome end is mainly orchestrated by the shelterin complex, and depends specifically on telomere length. Several observations note an increasing preference by telomerase for shorter telomeres [46,47]. Therefore, the classical conception of telomere length regulation is that the number of shelterin proteins inversely regulates telomerase recruitment and action by physically blocking the access of telomerase and hiding the 3′ end. However, recent studies propose a more complex regulation than a simple record of shelterin subunits. Indeed, a recent study described PINX1 as a physical interactor between TRF1 and TERT [48]. PINX1 is a telomerase inhibitor and is recruited by TRF1 at telomeres. The longer the telomere is, more TRF1 is present and more PINX1 is recruited, thus resulting in a stronger telomerase inhibition (Figure 1B). Moreover, TPP1 has been described to physically interact with telomerase, and to be essential for telomerase recruitment (Figure 1B) [49,50]. However, other studies suggest that this physical interaction is not enough for telomerase recruitment, and the cooperation of TIN2 with TPP1 is needed [51,52]. It remains unknown how the active site of telomerase captures the 3′ end, although several evidences point out POT1 into this telomere-telomerase engagement [44,53]. As a whole, further studies are needed to understand how shelterin regulates telomerase recruitment.

Once telomerase-telomere engagement has taken place, telomeric DNA aligns with TERC [33]. Then, telomerase adds de novo one telomere repeat at the 3′ DNA end [31,32,34,35] and, without dissociating from the telomere, repositions its RNA template to the new 3′ telomeric end. In human cells undergoing homeostatic telomere length maintenance, roughly 60 nucleotides are added in a processive manner to each telomere by a single binding and extension event [54,55]. This ability to add multiple telomeric repeats, known as repeat-addition processivity (RAP) [56], obliges dissociation of the product-template duplex without product dissociation from the enzyme [57]. Due to the structural flexible nature of telomerase, the newly synthetised telomeric DNA can be looped out [56]. Then, the RNA template is fully translocated and freed for the next round of telomeric repeat synthesis [56]. Other hypothesis suggests that the DNA strand remains bound to the telomerase active site, while the RNA template slips [58]. Then, the relocation of the 3′ RNA template would allow further DNA-RNA pairing [58].

Telomere elongation is promoted by POT1-TPP1 proteins, which function as a telomerase-stimulator [59], and proceeds until telomerase is released from telomeres. It seems likely that the distance between the 5′ end from the lagging strand and the 3′ end from the leading strand may itself lead to the inhibition or termination of telomerase processivity [60]. Indeed, the presence of a G-rich overhang can encourage structural conformations that displace telomerase [60]. In this sense, it has been described that the tight binding of the CST (CTC1-STN1-TEN1) complex to the G-strand telomeric substrate of telomerase, inhibits telomerase activity through primer sequestration (Figure 1B) [61,62]. Furthermore, the CST complex also represses telomerase by physically interacting with and inhibiting the POT1-TPP1 stimulator [61,62]. Depletion of POT-TPP1 increases human CST telomere association, suggesting that the two complexes compete for telomere overhang binding [61]. Once telomerase has been blocked, the CST complex may promote fill-in synthesis of the complementary C-strand by lagging strand polymerases (POL), as human CST subunits associate with and stimulate DNA polymerase-α RNA primase complex (POL-α) (Figure 1B) [61,62,63,64].

Although the great majority of cells rely on telomerase to add de novo telomere repeats, other mechanisms exist to regulate telomere length. Alternative lengthening of telomeres (ALT) is a recombination-based mechanism that elongates telomeres in around 15% of immortalised cell lines and human cancer cells [65,66,67]. The ALT-pathway uses telomeric sequences from another chromosome as a copy template to lengthen telomeres [66,68]. Direct demonstrations for inter-telomeric recombination events were obtained by the progressive increase in the number of tagged telomeres with population doublings (PDs) after targeting a DNA tag into a specific telomere [69]. In addition, ALT-cells are also recognised by their telomere length heterogeneity, with some chromosomes displaying very long telomeres and a subset lacking any discernible telomere signal at their end [70], as well as by the presence of extra-chromosomal linear [71,72] or circular [73,74] telomeric repeats. Yet another way to regulate telomere length is through telomere Trimming, an additional mechanism that also seems likely to contribute to normal telomere biology. Nevertheless, in contrast to telomerase or the ALT-pathway, telomere Trimming negatively regulates telomere length through the deletion of large segments of telomeric DNA in a single resolution event [75,76]. Although the underlying mechanism most likely involves HR-mediated removal of telomere loops in the form of t-circles, telomeres are not completely deleted, as telomere Trimming does not usually initiate a DNA damage response or result in telomere signal-free ends and chromosomal fusions [75]. The observation that extra-chromosomal t-circles were found to accumulate in telomerase-positive cancer cell lines following progressive telomere lengthening by exogenous telomerase activity [75] leads to suggesting that the prominent t-circles observed in ALT cells are, in fact, the product of telomere trimming counteracting extensive recombination-mediated telomere lengthening [77].

### Telomeres Shorten in Each Cell Division Cycle

As previously mentioned, telomerase is the main mechanism responsible for telomere elongation, however its expression is tightly regulated [78]. Whereas hTERC and the dyskerin complex are constitutively expressed in human tissues, hTERT is only expressed in the embryonic stem cells and in most adult stem cell compartments [33,34]. However, this is not sufficient to maintain telomere length [33,34]. Such circumstances pose a problem for most human cells, as the absent or reduced telomerase expression, leads telomeres to shorten every cell division cycle [79].

This “end replication problem” stems from the inherent asymmetry of how linear duplex DNA is copied during each cell division [80,81]. As the replisome, a complex DNA replication machine composed of multiple polymerases, moves along the chromosome, each strand is copied by one or more polymerases dedicated to that strand. Three polymerases, POL-α, POL-ε and POL-δ, are mainly responsible for DNA synthesis in eukaryotic cells [82]. Once the ds-DNA is opened by helicases, the POL-α-RNA primase complex synthesises small complementary RNA primers and a few DNA nucleotides, which are then elongated by the other polymerases. Although it is widely accepted that the asymmetric strand replication involves two different DNA polymerases, POL-ε and POL-δ, a recent paper suggest that POL-δ is the major DNA replicase [83]. Regardless of which polymerase/s acts, both strands grow at different rates due to the anti-parallel nature of DNA and the functional needs of the polymerase. The leading strand, synthesised in 5′-3′ direction, grows in a continuous manner from the RNA primer and becomes a blunt DNA end. This is not the case for the opposite strand, the lagging strand, which grows in a discontinuous manner from multiple Okazaki fragments that are finally connected by DNA ligase1. However, at the lagging-end telomere, removal of the terminal RNA primer results in non-blunt extremities and to the loss of a very small portion of chromosomal DNA. In human cells, the average rate of telomere shortening is 50–100 bp per population doubling [84].

In addition to the replication-associated shortening, telomeres are also reduced through enzyme processing in order to generate functional 3′ overhangs [64]. In mice, the resection of the C-rich strand depends on the Apollo nuclease [85,86,87] and exonuclease 1 (EXO1) (Figure 1C) [64]. Given that EXO1 preferentially degrades non-blunt ds-DNA in the 5′-3′ direction [88], the leading strand is initially resected by Apollo, which is recruited to the chromosome termini by TRF2 [64,85,87]. The emergence of ss-DNA in the leading strand induces TPP1-POT1a/b recruitment to the generated overhang that, in addition to protecting the ss-DNA blocks, blocks further Apollo processing [86]. Afterwards, EXO1 generates extended overhangs at both the lagging and the leading stands during late S/G2 phases [64] until its activity is probably inhibited by RIF1 via TPP1 [89]. Once overhang resection is blocked, POT1-TPP1 recruits the CST complex to promote filling in of the C rich strand, thus reducing the length of extended 3′ overhangs (Figure 1C) [64]. As a whole, telomere shortening occurs as consequence of incomplete DNA replication of the lagging-strand and C-strand processing at both the leading-end and the lagging-end telomeres in order to generate a G-overhang.

## 3. Telomere Protection and Cell Proliferative Boundaries

### 3.1. Excessive Telomere Erosion in Normal Cells Impairs Proliferation: Shortened Telomeres Elicit DDR

**Figure 2:**
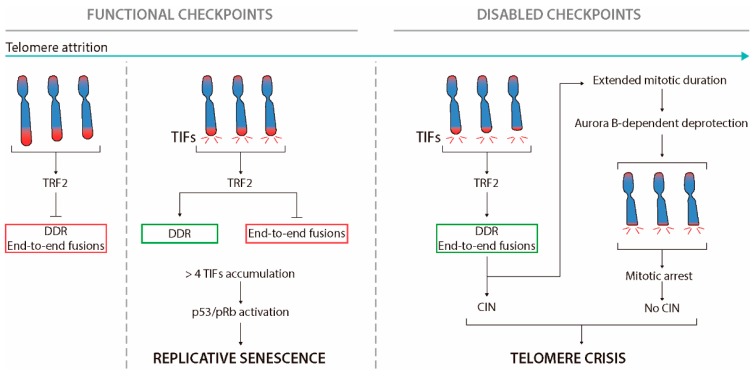
Telomere hypothesis of senescence and cancer. Telomere attrition occurs in cycling cells lacking telomerase activity or with reduced levels (green arrow). Long enough telomeres protect chromosome ends from DDR activation and end-to-end fusions (red box). After numerous PDs, few telomeres become too short and a local DDR signalling is activated at chromosome ends. At this stage, telomeres are not fully unprotected, as end-to-end fusions are prevented (red box) although the activation of the DDR (green box). Proliferation continues until a threshold of 4–5 TIFs directs cells to replicative arrest and senescence or apoptosis. In cells with functional checkpoints, excessive telomere shortening limits the proliferation of incipient cancer cells. Nevertheless, loss of the Rb and p53 tumour suppressors pathways allows cells to bypass cell cycle arrest in the presence of more than five telomere-induced foci (TIFs) per cell. During this lifespan extension period, further shortening leads to insufficient TRF2 retention at telomeres. These fully deprotected chromosome ends, in addition to activate the DDR, they promote the formation of end-to-end fusions (green box). It has been long speculated that the huge reorganisation of the genome associated to persistent telomere-dependent chromosomal instability (CIN) could allow the generation of pre-neoplastic cells if ultimately telomere length is stabilised. Otherwise, cells are directed to telomere crisis owing to massive genome remodelling, due to breakage-fusion-bridge (BFB) cycles, or/and to prolonged mitotic arrest and Aurora B-dependent TRF2 eviction from telomeres. Figure adapted from Arnoult and Karlseder 2015 [93].

In most tissues, excessive telomere shortening has been associated with a reduced cell proliferative capacity (Figure 2) [84]. The first evidence of this proliferative barrier was described by Hayflick and Moorhead in primary fibroblasts in vitro [79]. These authors observed that cultured fibroblasts possess a limited number of cell divisions, as after many cell doublings the cells lose their capacity to divide [79]. This maximum replicative potential, which occurs in different somatic cells and among different species, was called senescence [90]. The cells in this state, although metabolically active, enter a permanent cell growth arrest in G1 [79,91]. Senescence was then attributed to excessive telomere shortening, as the reactivation of telomerase compensated for telomere loss and extended cellular lifespan [92]. At present, other factors, besides telomere length, such as DNA damage, oxidative stress, modifications in chromatin, or activation of tumour suppressor proteins, are able to induce cellular senescence [91].

It is currently known that replicative senescence arises when progressive reduction of telomere length ends up with t-loop collapse and dysfunctional telomeres [94]. When shelterin levels are excessively reduced, the generation of the t-loop is compromised, as there is not enough TRF2 to remodel linear telomeric DNA. Consequently, telomeres shift towards an open deprotected state that activates a local DNA damage response (DDR), a signalling pathway associated with DSBs recognition and repair. The phosphorylated form of histone H2AX protein (γH2AX), which targets DSBs, also localises at dysfunctional telomeres in conjunction with other repair proteins, such as ATM, MDC1, 53BP1, or RAD17 [95,96]. The accumulation of these repair factors at telomeres are known as telomere dysfunction foci (TIFs) (Figure 2) [95].

Kaul et al. (2012) described that human fibroblasts accumulate spontaneous TIFs during cellular lifespan [97]. Cells keep dividing until they reach 4–5 TIFs, and above this threshold persistent telomere damage enforces cells to engage replicative senescence through p53-dependent signalling [97,98,99]. At this point, dysfunctional telomeres can recruit DDR factors, however they remain protected as at replicative senescence end-to-end fusions are not observed [97]. This ability to activate the DDR but to prevent telomeric fusions is thought to be mediated by the retention of some TRF2 molecules at chromosome ends [100,101]. In 2009, Karlseder’s Laboratory proposed that telomeres fluctuate through different protective states that rely on the abundance of TRF2 protein at chromosome ends, rather than to telomere length itself [100]. When telomeres are long enough to accommodate sufficient TRF2 to form a functional t-loop structure they remain in a full protected state. In senescence, telomeres are not fully unprotected, but remain in an intermediate-protection state in which the absence of t-loop generation leads to DDR activation, although telomeres retain enough TRF2 to prevent end-to-end chromosome fusions [97,100,101,102]. This DDR modulation relies on two independent domains of TRF2 [102]. Whereas the TRFH domain represses ATM activation at chromosome termini, preventing DDR and TIFs formation, the iDDR motif within TRF2 blocks non-homologous end-joining (NHEJ) obstructing 53BP1 accumulation and propagation of the DDR signals by inhibiting ubiquitin ligase RNF168 activity [102]. Therefore, the retention of some TRF2 at telomeres allows DDR signalling and the presence of TIFs in the absence of telomeric fusions. These TIFs, which remain in a fusion-resistant state, do not activate CHK2 kinase [99]. Consequently, cells do not succumb to the activation of the G2/M checkpoint, and progress through the cell cycle [99]. The resulting daughter cells containing the inherited TIFs will continue cycling until they reach the threshold of 4–5 TIFs that will direct cells to replicative senescence (Figure 2) [97].

### 3.2. Extended Lifespan by Tumour Suppressors Loss-of-Function

Studies with human cells have shown that the abrogation of p53 is not enough to circumvent replicative senescence, and pRb inactivation is also needed [103]. Therefore, both pathways act together to force and maintain cell cycle arrest and induce cellular senescence. Fully deprotected telomeres are only observed when inactivation or mutation of p53 and pRb pathways allows cells to escape from replicative senescence and to continue dividing until crisis, a state where most cells die [104]. During this second proliferative phase, telomeres become even shorter, reaching a length that cannot retain enough TRF2 to inhibit NHEJ [100] and subsequent end-to-end chromosome fusions (Figure 2) [105].

It is known that uncapped telomeres can initiate endless breakage-fusion-bridge (BFB) cycles and generate elevated levels of genome remodelling [106]. A common assumption is that the huge karyotype reorganisation of cells could trigger the massive cell death associated with crisis. Recently, Hayashi et al. (2015) suggested that the underlying molecular signal that triggers cell death at crisis is not a long-term process related to ongoing BFB cycles, but to exaggerated telomere deprotection upon mitotic arrest [107]. This model proposes that the presence of chromosome end-to-end fusions extends mitotic duration and activates an Aurora kinase B mediated dissociation of TRF2 from telomeres [107]. The cellular impact of Aurora dependent TRF2 eviction from telomeres is the amplification of the telomere damage, which ensures the correct DDR activation and leads to cell death immediately during mitotic arrest or in the subsequent G1 cell cycle phase [108]. This Aurora-dependent TRF2 dissociation was first observed in primary human fibroblasts following prolonged mitotic arrest, and was dependent on the time spent in mitosis (Figure 2) [108]. More recently, Hain and colleagues investigated the molecular mechanisms involved in the telomeric DDR induced by mitotic arrest [109]. They observed a reduction of γH2AX foci and increased levels of TRF2 at telomeres when cells were treated with caspase inhibitors, or when MCL-1 was over-expressed [109]. It is known that prolonged mitotic arrest promotes a time-dependent degradation of MCL-1, an anti-apoptotic member of BCL-2 family proteins, subsequent mitochondrial outer membrane permeabilisation, and the activation of the classical caspase-9/3/7 pathway that ends-up in endonuclease CAD/DFF40 switch on [110,111,112]. The additional observation that TRF2 depletion was inhibited by CAD endonuclease and DNA-PK inhibitors lends support to a model where the classical caspase pathway causes DNA damage at telomeres by producing DSBs and selectively activates DNA-PK. This promotes TRF2 loss, resulting in telomere deprotection and the formation of telomeric γH2AX foci, most likely by direct phosphorylation of H2AX by DNA-PK [109]. Thus, it appears that there is a response that selects against cells that fail to undergo chromosome segregation on schedule, and which are likely to produce daughter cells that carry aberrant chromosomes. However, an unresolved question is how exactly TRF2 is removed from telomeres, and whether the caspase-pathway and subsequent DNA-PK activation modulates Aurora B-dependent TRF2 depletion. However, specifically, in the case of telomere dysfunction, it remains to be established how the few fusions observed in crisis cells can trigger a prolonged mitosis arrest.

## 4. Dysfunctional Telomeres as a Source of Genome Instability and Cancer

### 4.1. Telomere Uncapping and Chromosome Instability

The ability of dysfunctional telomeres to promote genome instability originates in part, from their terminal chromosome location as activation of DDR at telomeres and subsequent repair activities results, primarily, in telomere fusion between distinct chromosomes or sister chromatids. Different studies in mouse and human cells suffering replicative telomere attrition have demonstrated that the chromosomes with the shorter telomeres are the first ones to become dysfunctional and to be involved in end-to-end fusions [113,114,115,116,117]. Similarly, telomere dysfunction, driven by shelterin modification, also results in end-to-end chromosome fusions [26]. Nevertheless, in this latter case, the dicentric chromosomes, or chromatids, do present telomere FISH signals at the fusion point when hybridised with a TTAGGG probe, as dysfunction is due to t-loop collapse and not to the shortening of the telomeric track [26].

In proliferating cells, chromosome end-to-end fusions due to telomere attrition can set BFB cycles in motion, a self-perpetuating mechanism that produces massive scrambling of the genome. When dicentric chromosomes or chromatids are pulled to opposite poles, they may lag at the cell equator, forming chromatin bridges between the two bulk chromatin-segregating complements. These anaphase bridges usually break during mitosis [118,119,120,121], and generate structural chromosome aberrations when the newly formed DSBs are repaired with either a broken end or an eroded telomere in the next generation [113]. In contrast, cells lacking TRF2 function did not show evidence for repeated BFB cycles, although end-to-end fusions are also formed [122]. Besides the limited anaphase bridge breakage, recent studies have determined that persistent chromatin bridges can also suffer extensive fragmentation, leading to chromothripsis, a mutational process in which multiple broken regions are haphazardly re-joined [123], as well as associated kataegis mutational hotspots [124]. This connection between chromothripsis and dysfunctional telomeres was observed in cells suffering induced telomere crisis through TRF2^ΔBΔM^ expression [124], and also highlighted by siRNA of TRF2 on p53 deficient RPE-1 cells [125]. Altogether diverse structural chromosome aberrations can originate from anaphase bridge breakage, including non-reciprocal translocations (NTRs), deletions, amplifications, new dicentric chromosomes [113,126,127], or hundreds of clustered genome rearrangements [128], which may result in loss of heterozygosity and cause oncogene amplification, amplification of oncogene containing regions and the loss of tumour suppressors.

However, breakage is not the only fate of anaphase bridges. It has been described that chromosomes with deprotected telomeres are more prone to missegregation than those with a non-critical telomere length, thus engendering aneuploid progeny [118]. The potential mechanism underlying chromosome bridge-induced aneuploidy is still unclear, although it may depend on the forces exerted by k-fibers [129]. The observation that k-fibers bound to bridge kinetochores shorten only slightly, and may even lengthen during anaphase, supports the notion that differential k-fiber dynamics may cause the bridged chromosomes to be segregated into the same daughter cell [129]. Alternatively, numerical chromosome aberrations appear when persistent chromatin bridges between segregating complements induce furrow regression and cytokinesis failure [130]. Likewise, whole genome duplication due to telomere uncapping has been described to arise in a chromatin bridge-independent manner [131]. Depletion of the shelterin proteins POT1a/b, or TRF2, in p53 defective mice, produced persistent telomeric DNA damage signalling that leads to the progressive accumulation of polyploid cells containing duplo- and quadruplo-chromosomes [103,131]. These cells experienced a prolonged G2 arrest owing to excessive damage and ATM- or ATR-pathways activation, but eventually skipped mitosis and re-entered the cell cycle in a polyploid state.

Collectively, it has been generally assumed that the autocatalytic nature of BFB cycles during telomere attrition induced crisis, massively scrambles the genome, yielding a wide range of lesions that threatens cell viability. Of note, cancer is an evolutionary disease in which the clonal nature has long been appreciated [132]. For Darwinian selection to occur, tumour cells may preserve the aptitude to maintain phenotypic heterogeneity through genomic instability [133]. Therefore, the karyotypic heterogeneity engendered through BFB-cycles may allow for the appearance of some rare advantageous mutations that would be selected and ultimately favour neoplastic progression. It has recently been proposed that telomere-driven crisis in human cells is not a long-term process that reorganises the genome, rather it is caused by generalised telomere deprotection during spontaneous mitotic arrest. Although the complete karyotype of the crisis cells was not established, telomere and centromere FISH showed only a few fusions events [107]. Therefore, it remains to be established how this observation reconciles with the generation of chromosomal instability (CIN) and the prevalent view of telomere-dependent CIN role in cancer onset and evolution.

### 4.2. Tumour-Promoting Effects of Telomere Dysfunction in the Mouse Not Yet Demonstrated in Humans

Studies to determine the in vivo effect of telomere dysfunction were first performed in mice that lacked the telomerase RNA component (*mTerc^−/−^*) [134,135]. Cytogenetic analysis of telomerase deficient cells after successive intercrossing of *mTerc^−/−^* animals showed an increase in end-to-end chromosome fusions, NTRs, and aneuploidy [134,136]. Moreover, 6th generation *mTerc^−/−^* mice showed shortened lifespan, ageing-associated pathologies, and multiorgan degeneration. These phenotypes were linked to the impaired proliferation of progenitor cells of highly proliferative tissues, due to the loss of cell viability and an increase in apoptosis [135,137,138]. The presence of extremely short telomeres activates the p53 pathway, which induces increased levels of p21^CIP1/WAF1^ and the inappropriate proliferation of damaged cells [139,140]. Consistent with these observations, *mTerc^−/−^* animals were resistant to cancer development [134,135]. Moreover, the induction of telomere dysfunction in mouse models relevant for cancer also did not denote a tumour-prone effect [141,142,143,144]. Strikingly, the abrogation of p53 function in *mTerc^−/−^* mice with dysfunctional telomeres enabled survival in the face of telomere dysfunction, and rescued many of the associated premature aging phenotypes, but at the expense of facilitating cellular transformation with MYC oncoprotein and GTPase Ras (RAS) [139]. Therefore, even although telomere-dysfunction was envisioned as a potent tumour suppressor mechanism, in the absence of functional checkpoints, it promoted genomic instability and initiated tumorigenesis. Similarly, animal studies where telomeres were rendered dysfunctional following conditional removal or hypomorphic mutations of shelterin proteins in combination with p53 mutations also lead to tumorigenesis [145,146,147,148]. However, most importantly, p53 mutations in late generation telomerase null mice produced a shift in the tumour spectrum towards epithelial cancers, the tumour type most frequent in aged humans, reinforcing a connection between telomeres and carcinomas [149]. In all, these findings demonstrate that telomere uncapping, either through loss of the G-rich overhang itself or by critically shortening the (TTAGGG)_n_ tract of DNA, can trigger, in the setting of p53 deficiency, the genome instability that promotes the development of epithelial cancers.

Telomere dysfunction activates and sustains the CIN needed to scramble the genome and promote incipient cancer cells, nevertheless these highly reorganised cells are inevitably directed to death, unless stabilisation of the chromosome ends takes place through the activation of telomerase or recombination mechanisms. Indeed, the formation and development of a small fraction of tumours in *mTerc^−/−^* mice, despite the complete absence of telomerase, was caused by ALT-recombination pathway activation [150]. The dual role of telomere dysfunction in murine carcinogenesis has been elegantly depicted using an inducible TERT expression system [151]. The conditional expression of telomerase after a lagging time period of telomere dysfunction, in a *p53/Pten*-null mice prostate cancer model, enabled the progression of aggressive metastatic tumours. Importantly, comparative oncogenomic analysis revealed numerous recurrent amplifications and deletions of relevance to human prostate cancer. Similarly, telomerase reactivation in T-cell lymphomas arising in *Atm^−/−^* mice, in the setting of telomere dysfunction led to full malignant progression, whereas telomerase extinction provoked slowdown of tumour growth [152]. Intriguingly, two tumour lines were able to resume growth, in the absence of telomerase function. Coincident with previous studies [150], these cell lines showed ALT-associated promyelocytic leukaemia bodies and extra-chromosomal telomere fragments, depicting the acquisition of alternative lengthening of telomeres.

In addition to the murine studies, mounting evidence supports a role for telomere dysfunction in human carcinogenesis. In human aging populations, cancer death is primarily due to carcinomas of the lung, liver, colorectal, stomach and breast [153]. These epithelial compartments undergo continual renewal through life, by suffering numerous cycles of proliferation and replacement. Although stem cells show active telomerase, their levels of expression are not sufficient to sustain telomere length in such compartments [154,155]. Therefore, the telomere attrition that should occur in these compartments, together with the fact that p53 mutations are observed in 20–30% of breast, 30–40% of lung, and over 40% of colorectal neoplasia [156,157], supports a permissive environment of telomere-induced genomic instability. Indeed, telomeres in human carcinoma are significantly shorter than their normal tissue counterparts [158], and telomere-to-telomere fusions have been detected in early stage breast tumours [159]. Collectively, all these data support the notion that in epithelial compartments, the combination of telomere shortening with checkpoint deficiencies could engender pre-neoplastic cells.

Besides the end-replication problem due to absent or reduced telomerase expression, recent studies have determined that mutations in the proteins belonging to the shelterin complex may lead to various malfunctions and ultimately also play a role in human tumorigenesis. Patients with early-stage chronic lymphocytic leukaemia (CLL) have an increased frequency of dysfunctional telomeres and telomere-to-telomere fusions are observed in advanced stages of the disease [160,161]. In agreement with a role of telomere-dysfunction in CLL, reduced expression levels of TRF1, RAP1 and POT1 [162], as well as TIN2 and TPP1 [161] have been detected. Furthermore, somatic mutations in POT1 account for 5% of CLL cases [163]. Of note, in addition to leukaemia, shelterin gene mutations have been described in melanoma [164,165], glioma [166] and sarcoma [167], thus indicating a broad potential relevance in non-epithelial oncogenesis.

Finally, numerous interactions between viral proteins and host telomere regulatory factors have been reported. Specifically, the Epstein–Barr virus (EBV) protein EBNA1 significantly increases the number of chromosomes with abnormal telomeres and decreases the amount of telomere-associated TRF2 through oxidative stress, but does not reduce the average telomere length or TRF2 levels in total cell lysates [168]. This is in line with the observed reduced levels of the shelterin components TRF1 and TRF2 at telomeres after the generation of reactive oxygen species [169]. Moreover, it was observed that the permanent expression of LMP1, another EBV oncoprotein, induces very short telomeres and telomere aggregates, as well as a significant downregulation of TRF1, TRF2 and POT1 at the transcriptional and translational level together with a disappearance of telomere-associated TRF2 spots, and the formation of abundant small TRF2 free telomeres [170]. Thus, the alteration of the normal host telomere structure and the generation of chromosome instability could be one of the mechanisms driving a variety of virus-induced lymphoid malignancies [171].

As a whole, although different mechanisms can modulate telomere homeostasis and drive aberrant telomeres, owing to the limiting lifespan factor of dysfunctional telomeres, the development of human neoplasia may be aborted by telomere-induced crisis long before lesions become macroscopic. Only those unstable cells able to reduce their CIN levels by stabilising their chromosome ends might further evolve and ultimately acquire a tumour phenotype. This supports the notion that approximately 80–90% of human cancers express telomerase and the remainder activate ALT-recombination-based mechanisms to cap chromosome ends [67,172].

## 5. Targeting Telomeres and Telomerase for Cancer Treatment

Given that telomerase expression is upregulated in the majority of carcinomas and soft tissue cancers, and that low levels of telomerase are observed in most normal tissues [173], several therapeutic strategies targeting telomerase have been developed to treat or fight cancer. The most prominent therapeutic strategy to block telomerase action is the use of antisense oligonucleotides. Imetelstat, also known as GRN163L, is an antisense oligonucleotide complementary to the template region of TERC [174], and is by far the most promising telomerase inhibitor. Several studies in vitro with human cancer cell lines have shown a strong inhibition of telomerase activity after GRN163L treatment [174,175]. Telomerase inhibition resulted in impaired cell growth and increased apoptosis, and of relevance, cell lines with critically short telomeres were the ones that were severely affected [174,175,176]. Moreover, persistent antisense oligonucleotide treatment also resulted in a decreased tumour take rate after engraftment of cancer cells pre-treated with Imetelstat [177]. Despite the great in vitro and in vivo potential of antisense oligonucleotides, clinical trials showed limited efficacy and nonspecific toxicity [174,178]. In addition, in addition, these therapies based on telomerase inhibitors required a long period of treatment to induce cell death. Another strategy to inhibit telomerase is the use of G-quadruplex (G4) stabilisers. G4 are secondary DNA structures rich in guanines which associate through hydrogen bonds to form planar structures that can stack and physically interfere with biological processes. Telomeres are G-rich sequences, therefore, it is not surprising to find G4 at telomeres, although they may form anywhere in the genome. Stabilisation of G4 at telomeres prevents DNA unfolding and inhibits telomerase access and subsequent elongation. However, short term G4 stabilisers treatment has very little effect on telomere length [179]. A second effect of G4 stabilisers is their capacity to displace shelterin proteins, resulting in telomere uncapping and DDR activation [180,181]. Indeed, after G4 ligand treatments, multinucleated cells and end-to-end fusions were observed to increase [179,180,182]. Although G4 stabilisers results in cell growth arrest in melanoma and brain tumour cells in vitro and in vivo [179,180,182,183], given that G4 structures are not exclusively found at telomeres, its stabilisation can induce off-target effects.

In addition to length regulation, shelterin proteins play essential roles in suppression of DNA damage signalling and inappropriate repair by homologous recombination (HR) and NHEJ at telomeres. Disruption of these protective functions occurs in ageing normal cells and can be achieved by genetic approaches. Telomere uncapping through shelterin modification precipitates telomere dysfunction and fast cell growth inhibition. One way to disrupt telomere protection is by the expression of mutant hTERC (Mt-hTERC) templates, which generates erroneous newly synthesised telomeric-strands that prevent shelterin binding and protection. After Mt-hTERC expression there were no changes on telomere length [184] and reduced TRF2 levels resulted in an increased number of γH2AX or 53BP1 foci at chromosome ends [185]. Mt-hTERC expression in immortalised hTERT fibroblast and in human cancer cell lines resulted in cell growth arrest, reduced viability and increased apoptosis [184,185,186,187]. Similarly, in vivo studies with human cells engrafted in mice resulted in the inhibition of tumour growth, increased apoptosis and minimal tumour angiogenesis [184,186]. Overall, Mt-hTERC therapies induced a cell response independent of telomere length but dependent on shelterin loss. Recently, targeted therapies to render telomeres dysfunctional through direct manipulation of shelterin proteins have been conducted. Targeting TRF2 through lentiviral vectors and RNA interference technologies inhibited the in vitro growth of primary human glioblastoma stem cells in addition to sensitising cells to temozolomide treatment [188]. Moreover, mice engrafted with the glioblastoma cells containing the TRF2 inhibiting lentivirus showed a significant extension of survival compared to those mice injected with unmodified glioblastoma cells [188]. These observances thus encouraged the use of modified shelterin for treating cancer patients. However, the toxicity of viral vectors is a major issue of concern when applying in vivo therapies in humans. Therefore, efforts have been made to design and synthesise chemical compounds that target specific shelterins. In this sense, a TRF2 permeable peptide that blocks its dimerisation domain has been developed and tested in vitro in HeLa cells [189]. Treatment with the compound for 24 h resulted in an increased number of TIFs and DDR activation at chromosome ends that was compatible with intermediate-state telomeres, as there was an almost complete lack of 53BP1 recruitment as well as undetectable end-to-end fusions [189]. Besides TRF2 studies, therapeutic inhibition of TRF1 impaired the growth of *p53*-deficient *K-Ras^G12V^*-induced lung cancer in the mouse independently of telomere length [190]. This decrease in proliferation was accompanied by the presence of telomeric DNA damage, apoptosis and G2 arrest already in the first mouse generation [190]. Recently, brain-specific *Trf1* genetic deletion in glioblastoma multiforme (GBM) mouse models, inhibited cancer initiation and progression by increasing telomeric DNA damage [191]. Moreover, TRF1 chemical inhibitors mimicked these effects in human GBM cells and also blocked tumour sphere formation and tumour growth in xenografts from patient-derived primary glioma stem cells [191]. As a whole, targeting telomeres throughout shelterin inhibition has been demonstrated to be an effective therapeutic strategy for glioblastoma and should probably be a valuable therapy for many other cancer types.

## 6. Conclusions

In recent years, much has been learned about the role of telomeres in cancer. Cancer cells require multiple mutations to become malignant, and it is clearly accepted that chromosome instability (CIN) should be a driver for this mutator phenotype. Telomere-dysfunction has proved to be an inducer of CIN when impaired checkpoints allow for cell-cycle progression of cells carrying short telomeres. However, telomere-dependent CIN must be transient to avoid the detrimental effect of telomere dysfunction. Indeed, mouse studies have evidenced the need for a period of telomere instability to remodel the genome but afterwards, telomerase or recombination mechanisms must be upregulated to lessen the excessive genome scrambling that would direct cells to crisis. Giving rise to the recent observation suggesting that cell death during telomere crisis is not due to rampant genome instability, but to the presence of excessively deprotected telomeres. The next exciting field will be to understand whether the degree of telomere dysfunction also modulates distinct cellular responses. Moreover, continued investigation on how telomere-dysfunction can promote tumour initiation in human cells will provide a greater understanding and clues to telomere biology in cancer development and treatment.
